# Socio-Demographic Factors Affect the Prevalence of Hematuria and Proteinuria Among School Children in Hualien, Taiwan: A Longitudinal Localization-Based Cohort Study

**DOI:** 10.3389/fped.2020.600907

**Published:** 2020-12-10

**Authors:** Ming-Chun Chen, Jen-Hung Wang, Jui-Shia Chen, Yung-Chieh Chang, Rong-Hwa Jan, Shang-Hsien Yang, Shao-Yin Chu, Pei-Chun Lai, Chia-Hsiang Chu, Ching-Feng Cheng, Yu-Hsun Chang

**Affiliations:** ^1^Department of Pediatric, Hualien Tzu Chi Hospital, Buddhist Tzu Chi Medical Foundation, Hualien, Taiwan; ^2^School of Medicine, Tzu Chi University, Hualien, Taiwan; ^3^Department of Medical Research, Hualien Tzu Chi Hospital, Buddhist Tzu Chi Medical Foundation, Hualien, Taiwan; ^4^Evidence-Based Medicine Center, Department of Medical Education, Hualien Tzu Chi Hospital, Buddhist Tzu Chi Medical Foundation, Hualien, Taiwan; ^5^Department of Pediatric, Taipei Tzu Chi Hospital, Buddhist Tzu Chi Medical Foundation, Taipei, Taiwan; ^6^Department of Pediatric, National Taiwan University Hospital, Hsinchu, Taiwan

**Keywords:** body mass index, children, chronic kidney disease, hematuria, mass urinary screening, proteinuria

## Abstract

**Objective:** Child hematuria/proteinuria is a risk factor for chronic kidney disease (CKD) in later life, and mass urinary screening could detect asymptomatic glomerulonephritis at an early stage. This study aimed to evaluate the longitudinal prevalence of hematuria/proteinuria and its association with socio-demographic factors among school children in Hualien, Taiwan.

**Methods:** The study cohort consisted of first and fourth graders enrolled from 2008 to 2015 in Hualien. We combined the data from two consecutive health examinations to ensure the validity of the body mass index (BMI), urbanization, proteinuria, and hematuria grouping. Prevalence and health status differences between sex, age, BMI, and urbanization level were examined.

**Results:** A total of 16,990 students within the same BMI and urbanization categories were included during the study interval. The prevalence of persistent hematuria was 1.0%. Fourth graders (odds ratio OR: 1.68, *p* = 0.002), girls (OR: 1.48, *p* = 0.014), and students from suburban/rural areas (OR: 1.99, and OR: 4.93, respectively; both *p* < 0.001) demonstrated higher hematuria risk. The prevalence of proteinuria was 0.2%. Fourth graders (OR: 4.44, *p* < 0.001) and students in suburban areas (OR: 0.27, *p* = 0.031) were associated with persistent proteinuria. After stratifying by age, the significant association remained. A higher risk of proteinuria was noted in underweight subjects (OR: 2.52, *p* = 0.023) among the fourth-grade students.

**Conclusion:** The prevalence of hematuria/proteinuria in Hualien was higher than the average reported for Taiwan. Hematuria/proteinuria was significantly associated with sex, age, BMI, and urbanization. Our longitudinal results can provide information for future pediatric CKD prevention in Taiwan.

## Introduction

Chronic kidney disease (CKD) is currently a major global health problem. Not only can CKD progress to end-stage renal disease (ESRD), it is also a major risk factor for cardiovascular disease in later life (1). In Taiwan, the annual incidence of pediatric ESRD was reported to be 8.12 cases per million of age-matched population (2), with hematuria and proteinuria being the most common manifestations of pediatric CKD at diagnosis (3). Early aggressive treatment of patients with nephrotic-range proteinuria and combined hematuria-proteinuria could delay CKD progression (4). Asymptomatic glomerulonephritis (GN), manifesting as urinary abnormalities, can be detected via the school mass urinary screening (MUS) system, and MUS programs have been established for early detection of GN in Asian countries such as Japan, Korea, Singapore, and Taiwan, where GN is frequently the primary cause of ESRD (4–7). Dipstick screening has been widely used as a simple and inexpensive method for detecting urinary abnormalities and has subsequently promoted public awareness of CKD (5, 7–9).

Socio-demographic factors have been shown to have a significant impact on CKD progression (10, 11). Adult studies have revealed that the prevalence of CKD is highest among low to middle-income countries (10), and Fraser et al. have also reported that CKD and proteinuria are associated with patient socioeconomic status (11). Hualien County, which is located in the East of Taiwan, has the largest aboriginal population (25% of all residents) and the second least urbanized county in Taiwan. From 1991 to 1998 in Taiwan, 0.3% of screened students had an abnormal urinalysis. Among them, 7.4% of these students had heavy proteinuria, with increased risks of developing hypertension and nephritis (5). Notably, students in Hualien had the third-highest 4-year prevalence of heavy proteinuria in Taiwan at that time (12). Furthermore, the prevalence of metabolic syndrome in junior high school students in East Taiwan was 5.4%, which was the second-highest percentage in Taiwan in 2010. The prevalence of increased low-density lipoprotein (8.0%) and decreased high-density lipoprotein (8.6%) in the blood is highest among children in East Taiwan as compared with Taiwan in general (13). These epidemiology studies, therefore, imply that children in Hualien may suffer from renal damage and an increased incidence of abnormal urinalysis.

Since 2007, health examinations, including urinalysis, have been provided for all grades 1, 4, and 7 school children in Hualien by the same tertiary center. Our prior studies have reported that the overall prevalence of hematuria and proteinuria among Hualien school children increased gradually between 2010 and 2013 (3.8 vs. 4.1% with hematuria and 2.4 vs. 5.7% with proteinuria, respectively) (13, 14). However, little information is available regarding the influence of socio-demographic factors on the same participant after long-term follow-up, or on the possible risk stratification of these students in Taiwan. Furthermore, a recent study also revealed that childhood socioeconomic status is associated with adult CKD progression (15). Therefore, the objective of the present study was to evaluate the longitudinal prevalence of hematuria/proteinuria and its association with different socio-demographic factors, including sex, age, body mass index (BMI), and level of urbanization, in school children within Hualien, Taiwan.

## Materials and Methods

### Subjects

**Figure 1:**
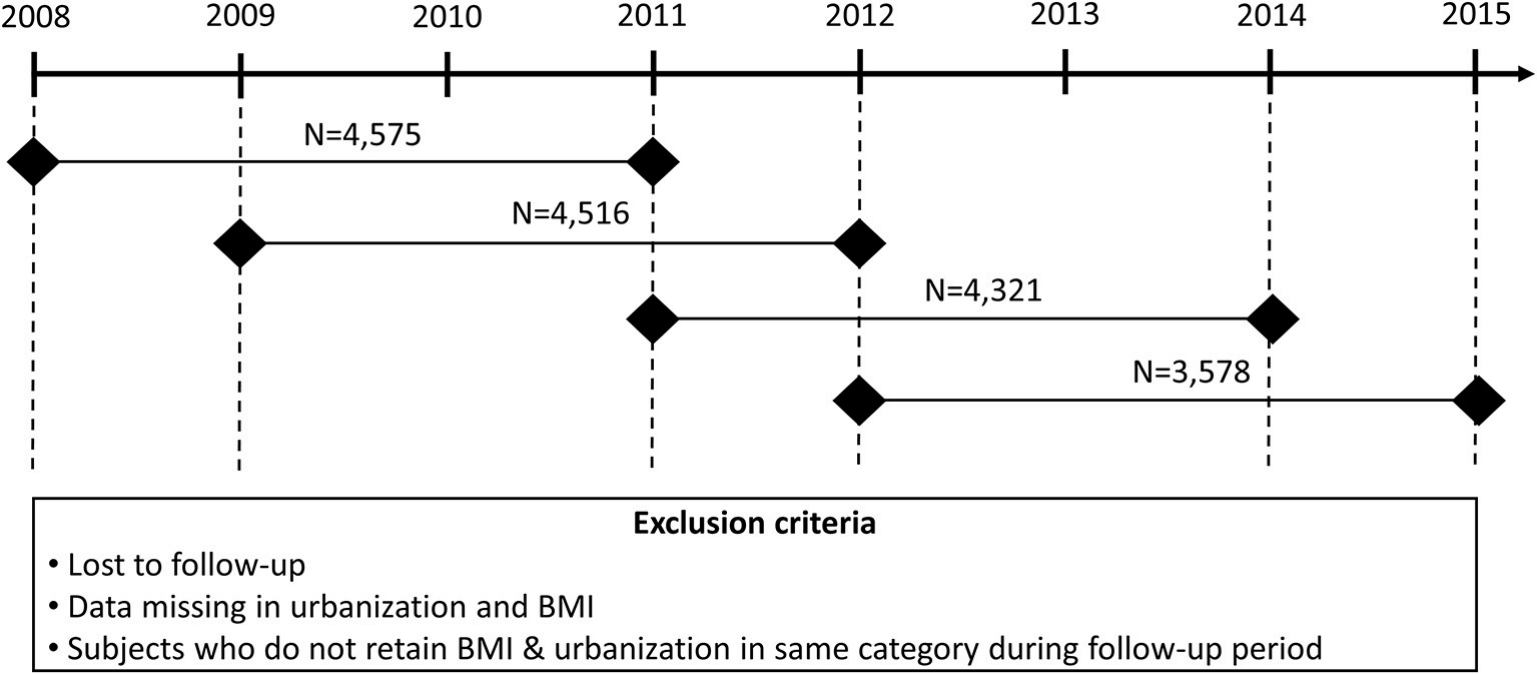
The study cohort consisted of first and fourth graders in 2008, 2009, 2011, and 2012. We combined the data from two consecutive health examinations and urine analyses to ensure the validity of the BMI group, level of urbanization, proteinuria, and hematuria.

Health examination and MUS data were routinely collected for the same students in his or her first (aged 7–8 years), fourth (aged 10–11 years), and seventh graders (aged 13–14 years) in Hualien County, Taiwan. Thus, we enrolled first and fourth graders in 2008, 2009, 2011, and 2012 to form the cohort. Consecutive health examination and MUS data after 3 years were also included to assure BMI, urbanization, proteinuria, and hematuria status. We compared the same students within the 3-year intervals in grades 1–4 or grades 4–7 to evaluate the risk factors associated with the longitudinal progression of proteinuria and hematuria. The study period was divided into four 3-year epochs: epoch 1 (2008–2011), epoch 2 (2009–2012), epoch 3 (2011–2014), and epoch 4 (2012–2015). A flow chart detailing our study design is depicted in Figure 1. A total of 24,788 students from 25 junior high schools and 105 elementary schools, with complete follow-up data, were enrolled in this longitudinal study. Students with the same BMI and urbanization category within these 3-year intervals (*n* = 16,990, 68.5% of the total population) were included in the analysis. Informed consent was obtained from the students' parents or guardians. The study was approved by the Hualien County Government Education Bureau and the Protection of Human Subjects Institutional Review Board of Tzu Chi University and Hospital (REC No.: IRB101-125 and IRB105-52-B).

### Measurements and Procedures

This study evaluated the longitudinal prevalence of hematuria and proteinuria and its association with different socio-demographic factors among school children in Hualien, Taiwan in two study periods. Risk factors believed to be associated with these abnormal urinalyses in school children, including sex, age, BMI, and urbanization levels, were also recorded. Follow-up measurements were performed 3 years after baseline testing at the elementary or junior high schools for all available children. The children were instructed to empty the bladder completely the night before sample collection. A midstream first-morning urine specimen was collected the following day and was examined with the dipstick method to detect hematuria and proteinuria as previously described (13, 16). Leukocyte and nitrite levels were also recorded for later adjustment for abnormalities indicating urinary tract infection. For both isolated hematuria and proteinuria, positive reactions were defined as persistent 1+ (25 red blood cells/L and protein 30 mg/dL for hematuria and proteinuria 1+, respectively) or higher in both baseline testing and follow-up measurements during the 3-year interval (8). Children were categorized as underweight, normal, or overweight/obese according to the age-gender-specific Taiwanese national BMI reference values for children and adolescents, as reported by the Ministry of Health and Welfare in Taiwan (17). The population density (people per square kilometer, km^2^) was used to determine the urbanization level. According to the population densities of residential areas, urbanization levels were divided into the following three groups: urban (>500 people/km^2^), suburban (15–500 people/km^2^), and rural (<15 people/km^2^) (18). Based on this categorization, three (23%) of the included areas were defined as urban, seven (54%) as suburban, and three (23%) as rural.

### Statistical Analysis

Data were analyzed using SPSS for Windows (version 21; SPSS Inc., Chicago, IL, USA). Descriptive statistics for hematuria and proteinuria are presented as absolute numbers and percentages. A Chi-square test was performed to identify parameters that were significantly associated with BMI. Logistic regression models were used to analyze associations between the prevalence of hematuria/proteinuria and socio-demographic factors. Crude and adjusted odds ratios, and 95% confidence intervals, were calculated. A *p* < 0.05 was defined as statistically significant.

## Results

Demographic information on the 16,990 students accepting examination during the four study epochs (2008–2011, 2009–2012, 2011–2014, and 2012–2015) is presented in Table 1. The overall prevalence of persistent hematuria and proteinuria was 1% (164/16,990), and 0.2% (42/16,990), respectively. Of the enrolled students, 47.0% were first-graders and 52.2% were male. Of the students with an unaltered urbanization level throughout the study period, 72.4, 22.9, and 4.8% of children lived in urban, suburban, or rural areas, respectively. We analyzed students that remained in the same BMI group throughout the study period; 64.1% had a normal BMI, 10.9% were underweight, and 25.0% were overweight/obese.

**Table 1:** Demographic information among students accepting physical examination (*n* = 16,990).

	**Period**				
**Variable**	**2008–2011**	**2009–2012**	**2011–2014**	**2012–2015**	**Total**
*N*	4,575	4,516	4,321	3,578	16,990
**Initial school grade**					
1	2,231 (48.8%)	2,068 (45.8%)	2,025 (46.9%)	1,654 (46.2%)	7,978 (47.0%)
4	2,344 (51.2%)	2,448 (54.2%)	2,296 (53.1%)	1,924 (53.8%)	9,012 (53.0%)
**Sex**					
Male	2,417 (52.8%)	2,368 (52.4%)	2,175 (50.3%)	1,866 (52.2%)	8,826 (51.9%)
Female	2,158 (47.2%)	2,148 (47.6%)	2,146 (49.7%)	1,712 (47.8%)	8,164 (48.1%)
**BMI patterns**					
BMI remaining normal	3,191 (69.7%)	2,712 (60.1%)	2,992 (69.2%)	1,988 (55.6%)	10,883 (64.1%)
BMI remaining underweight	170 (3.7%)	781 (17.3%)	178 (4.1%)	727 (20.3%)	1,856 (10.9%)
BMI remaining overweight/obese	1,214 (26.5%)	1,023 (22.7%)	1,151 (26.6%)	863 (24.1%)	4,251 (25.0%)
**Urbanization**					
Urban	3,300 (72.1%)	3,231 (71.5%)	3,108 (71.9%)	2,660 (74.3%)	12,299 (72.4%)
Suburban	1,052 (23.0%)	1,106 (24.5%)	997 (23.1%)	728 (20.3%)	3,883 (22.9%)
Rural	223 (4.9%)	179 (4.0%)	216 (5.0%)	190 (5.3%)	808 (4.8%)
**Hematuria**					
No	4,519 (98.8%)	4,462 (98.8%)	4,295 (99.4%)	3,550 (99.2%)	16,826 (99.0%)
Yes	56 (1.2%)	54 (1.2%)	26 (0.6%)	28 (0.8%)	164 (1.0%)
**Proteinuria**					
No	4,570 (99.9%)	4,509 (99.8%)	4,311 (99.8%)	3,558 (99.4%)	16,948 (99.8%)
Yes	5 (0.1%)	7 (0.2%)	10 (0.2%)	20 (0.6%)	42 (0.2%)

Data are presented as absolute numbers and percentages.

^*^p < 0.05 was considered statistically significant.

*BMI, body mass index*.

To clarify whether abnormal urinalysis was associated with a higher risk of these socio-demographic factors, we performed multiple logistic regression analysis. The results are summarized in Table 2. Female sex, older age, and habitation in suburban/rural areas were identified as risk factors for persistent hematuria. Girls had a significantly higher odds ratio (1.48) of hematuria as compared to boys (*P* = 0.014). Students enrolled in the fourth grade had a significantly higher odds ratio (1.68) of hematuria as compared to those enrolled in the first grade (*P* = 0.002). Students living in suburban and rural areas had significantly higher odds ratios (1.99 and 4.93, respectively) of hematuria as compared to those living in urban areas (both *P* < 0.001).

**Table 2:** Factors associated with a higher risk of abnormal screening items (*n* = 16,990).

**Variable**	**Hematuria**				**Proteinuria**			
	**Crude**		**Adjusted**^****a****^		**Crude**		**Adjusted**^****a****^	
	**OR (95% CI)**	***p*-value**	**OR (95% CI)**	***p*-value**	**OR (95% CI)**	***p*-value**	**OR (95% CI)**	***p*-value**
**School grade**								
1	1		1		1		1	
4	1.42 (1.04, 1.95)	0.028^*^	1.68 (1.21, 2.34)	0.002^*^	4.44 (1.97, 10.00)	<0.001^*^	4.62 (2.02, 10.56)	<0.001^*^
**Sex**								
Male	1		1		1		1	
Female	1.46 (1.07, 1.99)	0.018^*^	1.48 (1.08, 2.03)	0.014^*^	1.19 (0.65, 2.18)	0.574	1.16 (0.63, 2.14)	0.628
**Urbanization**								
Urban	1		1		1		1	
Suburban	1.98 (1.41, 2.79)	<0.001^*^	1.99 (1.41, 2.80)	<0.001^*^	0.26 (0.08, 0.83)	0.023^*^	0.27 (0.08, 0.89)	0.031^*^
Rural	4.11 (2.58, 6.55)	<0.001^*^	4.93 (3.04, 8.02)	<0.001^*^	0.82 (0.20, 3.42)	0.788	1.58 (0.37, 6.78)	0.539
**BMI pattern**								
BMI remaining normal	1		1		1		1	
BMI remaining underweight	0.70 (0.38, 1.27)	0.234	0.79 (0.43, 1.45)	0.450	2.21 (1.02, 4.75)	0.044^*^	2.13 (0.98, 4.60)	0.055
BMI remaining Overweight/obese	1.30(0.92, 1.82)	0.133	1.36 (0.97, 1.91)	0.077	0.96 (0.45, 2.07)	0.917	0.92 (0.43, 1.99)	0.839

a Adjusted for school grade, sex, urbanization, and BMI pattern. BMI, body mass index; CI, confidence interval; OR, odds ratio.

* *p < 0.05 was considered statistically significant after logistic regression analysis*.

In proteinuria risk factor studies, older age and living in suburban areas were associated with persistent proteinuria. The older students (fourth graders) had a significantly higher odds ratio (4.44) of proteinuria as compared to the younger ones (first graders) (*p* < 0.001). Students living in suburban areas had significantly lower odds ratios (0.27) of proteinuria than those living in the urban area (*P* = 0.031).

A positive correlation between BMI and hematuria was observed, although underweight and overweight/obese students did not have a significantly lower and higher odds ratio of hematuria as compared to that in the normal group. Interestingly, a negative correlation between BMI and proteinuria was noted throughout the study. Students who were overweight/obese had a lower odds ratio (0.92), and underweight children tended to have a higher odds ratio (2.13) of proteinuria as compared to those who remained at a normal weight, although this was not statistically significant (*P* = 0.839 and 0.055, respectively).

To further clarify the influence of age among these socio-demographic risk factors for hematuria or proteinuria, subgroup analysis was performed after stratifying by age (the results are shown in Table 3). Among first grade students, differences between sex and urbanization levels were observed. Female students had a significantly higher risk of persistent hematuria than male students, whereas students living in suburban/rural areas had a significantly higher risk of persistent hematuria than students living in urban areas. The difference in urbanization remained only in fourth-grade students. When evaluating risk factors related to proteinuria, there was no statistically significant difference between sexes, urbanization levels, or BMI among first graders. However, among the fourth graders, underweight students demonstrated a significantly higher odds ratio (2.52) of proteinuria compared with students who remained at a normal weight (*P* = 0.023).

**Table 3:** Factors associated with a higher risk of abnormal screening items, stratified for grade (*n* = 16,990).

**Stratification**	**Variable**	**Hematuria**				**Proteinuria**			
		**Crude**		**Adjusted**^****a****^		**Crude**		**Adjusted**^****a****^	
		**OR (95% CI)**	***p*-value**	**OR (95% CI)**	***p*-value**	**OR (95% CI)**	***p*-value**	**OR (95% CI)**	***p*-value**
First Graders (*N* = 7,978)	**Sex**								
	Male	1		1		1		1	
	Female	1.67 (1.01, 2.78)	0.046^*^	1.72 (1.03, 2.86)	0.038^*^	2.74 (0.53, 14.15)	0.228	3.01 (0.58, 15.68)	0.190
	**Urbanization**								
	Urban	1		1		1		1	
	Suburban	2.64 (1.47, 4.76)	0.001^*^	2.65 (1.47, 4.77)	0.001^*^	0.00 (NA)	0.988	0.00 (NA)	0.987
	Rural	6.48 (3.50, 12.01)	<0.001^*^	6.38 (3.43, 11.87)	<0.001^*^	1.41 (0.17, 11.71)	0.752	1.26 (0.15, 10.51)	0.830
	**BMI pattern**								
	BMI remaining normal	1		1		1		1	
	BMI remaining underweight	0.75 (0.30, 1.91)	0.548	0.93 (0.36, 2.38)	0.876	0.00 (NA)	0.992	0.00 (NA)	0.991
	BMI remaining overweight/ obese	1.30 (0.75, 2.28)	0.352	1.39 (0.79, 2.44)	0.254	2.17 (0.49, 9.71)	0.311	2.39 (0.53, 10.79)	0.256
Fourth graders (*N* = 9,012)	**Sex**								
	Male	1		1		1		1	
	Female	1.33 (0.90, 1.98)	0.154	1.36 (0.91, 2.02)	0.132	1.01 (0.52, 1.96)	0.981	0.97 (0.50, 1.90)	0.936
	**Urbanization**								
	Urban	1		1		1		1	
	Suburban	1.75 (1.15, 2.68)	0.010^*^	1.73 (1.13, 2.64)	0.012^*^	0.32 (0.10, 1.05)	0.060	0.34 (0.10, 1.10)	0.072
	Rural	3.52 (1.40, 8.88)	0.008^*^	3.48 (1.37, 8.80)	0.009^*^	1.40 (0.19, 10.33)	0.741	1.56 (0.21, 11.57)	0.666
	**BMI pattern**								
	BMI remaining normal	1		1		1		1	
	BMI remaining underweight	0.65(0.30, 1.43)	0.289	0.71 (0.32, 1.56)	0.390	2.59 (1.18, 5.70)	0.018^*^	2.52 (1.14, 5.57)	0.023^*^
	BMI remaining overweight/ obese	1.25 (0.82, 1.92)	0.299	1.33 (0.86, 2.04)	0.197	0.69 (0.28, 1.73)	0.430	0.70 (0.28, 1.75)	0.443

a Adjusted for school grade, sex, urbanization, and BMI pattern. OR, odds ratio; CI, confidence interval.

* *p < 0.05 was considered statistically significant after logistic regression analysis*.

## Discussion

The major findings from our longitudinal school children's urinary screening analysis are summarized as follows: female sex, older age, and habitation in suburban/rural areas were identified as significant risk factors for persistent hematuria. Older age was also identified as a risk factor for persistent proteinuria. After stratifying by age, similar socio-demographic risk factors were identified for persistent hematuria and proteinuria in first-grade students. However, a significantly higher risk of persistent hematuria was still noted among students living in suburban/rural areas, and a higher risk of persistent proteinuria was noted in underweight students who were older (fourth graders).

The measured prevalence of pediatric hematuria or proteinuria varies depending on different study methods, ethnic background, or geographic region (4, 5, 16, 19). Using the dipstick method in the urine specimen from the first screen, the prevalence of hematuria and proteinuria was 6.8 and 1.2%, respectively, in a screening program of 12-year-old school children in Singapore (19). In Taiwan, an epidemiological study revealed that the most common manifestations at diagnosis in children with CKD were proteinuria (41%) and hematuria (28%), with even higher rates of proteinuria (68.2%) and hematuria (44.1%) when GN is the underlying etiology of CKD (3). Our present study has revealed that the overall prevalence of persistent hematuria and proteinuria in Hualien County were 1 and 0.2%, respectively, which were higher than those of a previous study analyzing school students in Taiwan from August 1999 to January 2000 (20), suggesting that the incidence of hematuria and proteinuria have been gradually increasing in recent years.

A previous MUS study of school children in Taiwan revealed that girls were affected by proteinuria more often than boys (12). This contrasts with the present study in which we found no sex differences in students with proteinuria. However, we found that female students had a significantly higher risk of hematuria than male students. A study in Saudi Arabia also revealed that hematuria is more frequently observed in healthy adolescent girls, with a female to male ratio of 1.5:1, and without a significant correlation with ethnicity or nationality (21). Also, older female students had a higher prevalence of hematuria in the present study, which is consistent with previous reports (13, 16). Although this phenomenon may correlate with the possibility of menstrual blood contamination, persistent false positives due to menstrual blood contamination are less likely within our 3-year interval follow-up. Furthermore, the prevalence of GN is higher in Asian countries than in other geographic regions and is frequently the primary cause of ESRD as mentioned above (22). A recent study by Chiou et al. also confirmed that IgA nephritis and lupus nephritis are the most important primary and secondary GN, and the Taiwan Pediatric Renal Collaborative Study identified it as the most common disease in patients with CKD due to nephritis (37.2%) (3, 5). In Taiwan, pediatric CKD patients with nephritis were older (average age at diagnosis: 10.2 years), were more likely to be female (54.6%), had a higher prevalence of hematuria (44.1%) at diagnosis, and had a higher prevalence of autoimmune diseases (3). Furthermore, after further age-sex specific analyses, our previous cross-sectional study of students in Hualien also showed that seventh-grade female adolescents had the highest prevalence of hematuria and proteinuria compared with other students (13). Results from these studies indicate that females may be more prone to abnormal urinalysis due to autoimmune-mediated GN, and this may have contributed to the difference in hematuria distribution between sexes in our study population.

Proteinuria has been shown to be a significant risk factor for pediatric CKD progression (23). Our present study reported a significantly high prevalence of proteinuria in older students. Not surprisingly, the prevalence of renal disease increased gradually with age, and many studies have reported similar findings (12, 13, 16). An epidemiological study in Taiwanese pediatric patients with CKD due to nephritis also revealed an even higher prevalence of proteinuria than hematuria at diagnosis. Furthermore, the median age of nephritis-induced CKD patients was 10.2 years, which is similar to our findings in the fourth graders (10–11 years) (3). Although many studies have demonstrated that an increased BMI is significantly associated with proteinuria risk (21, 24–27), our present study revealed that the prevalence of proteinuria was not elevated in persistently overweight or obese students. This result may be affected by our study population, since prior studies stated that the correlation between BMI and CKD was mainly attributed to the subcategory of morbid obesity, with a BMI >35 kg/m^2^ (28). Most students classified as overweight/obese in our study did not fit the criteria for morbid obesity (only 63 (0.4%) students were morbidly obese); thus, no associations were identified between proteinuria prevalence and BMI in our 3-year interval study period. Moreover, the progression of renal damage increased with age, but most of the study population were younger elementary school students with less comorbidity-induced renal damage. Therefore, future long-term follow-up of these overweight/obese students, especially morbidly obese individuals, is necessary.

Conversely, our study revealed that persistently underweight students had an increased prevalence of proteinuria and this became statistically significant with increasing age, as confirmed by further subgroup analysis stratified for age. There is increasing evidence that the association between proteinuria and BMI shows a clear U-shape trend, as well as remarkable differences between the sexes (29). Previous studies have shown that low BMI is associated with proteinuria (30), and that proteinuria and decreased BMI are independent risk factors for developing CKD and ESRD (31, 32). Our findings are consistent with a recent study by Lin et al., which found that students with CKD with albuminuria in a Taiwanese elementary school had significantly lower body weights than students without CKD (33). Although the relationship between low BMI and pediatric proteinuria is still uncertain, decreasing BMI could be due to muscle wasting, malnutrition, or volume depletion, which can aggravate CKD progression and subsequently increase the severity of proteinuria (32). In addition, a larger odds ratio of proteinuria was particularly prominent in the younger subjects with the lowest BMI, and this relationship was not found in the higher BMI group, suggesting the presence of more GN than postural proteinuria or nephrosclerosis in the younger subjects with the lowest BMIs (29).

Our present study showed that persistent hematuria was more prevalent in children living in suburban and rural areas than in those living in urban areas. After performing age-specific analyses, this phenomenon remained. In Taiwan, low levels of urbanization often accompany poor sanitary conditions, increased toxins exposure, and reduced medical resources. Although herbal remedies, such as aristolochic acid, have been prohibited since November 2003 in Taiwan, these herbs may be inappropriately used in the less urbanized area and could increase the risk of renal damage (34). Furthermore, Hualien water contains a high amount of calcium, and the drinking water is classified as moderately hard to hard according to the reference data from Taiwan Water Supply Corporation (13). Consuming unsanitized water with elevated calcium levels may increase the risk of hypercalciuria-induced hematuria in the students living in more rural areas (12, 35).

The strength of this study is that, to the best of our knowledge, it is the first location-based, longitudinal cohort study in Taiwan evaluating the risk factors of hematuria and proteinuria in school children with adjustment for socio-demographic factors. In our study, we analyzed the same children using a 3-year interval follow-up, and most of the school-aged children of Hualien County live in the same urbanization area. The distribution of urbanization, after comparing four study epochs, showed no statistical significance; hence, this follow-up study represents a true longitudinal evaluation of CKD risk in these students without bias resulting from loss to follow-up of specific groups. Secondly, students within the same BMI classification throughout the study period were included for analysis, and using age-specific BMI cutoffs allowed the variability in age at measurement to be taken into account. Thirdly, health examination data were collected from 2008 to 2015 for longitudinal analysis, and thus, the directionality cause-effect relationship between hematuria/proteinuria and different socio-demographic factors was more evidenced than in the previous cross-sectional study (13). There is little information on socio-demographic factors influencing CKD progression in the pediatric population. A previous study in Australia revealed that socioeconomic and geographical disadvantages had no significant influence on hematuria and proteinuria among 2,266 Australian indigenous and non-indigenous children (36). Our large cohort study consisting of 16,990 students after a 6-year follow-up revealed that socio-demographic factors had a significant association with hematuria and proteinuria, although the results are not in line with those of the Australian's study. Our results could be helpful for providing preventive measures in school children, before they may develop kidney disease not only in Taiwan but also in other countries.

Our current study had the following limitations. First, several risk factors, such as local toxins, use of herbal remedies, and genetic effects which may influence the occurrence of hematuria and proteinuria are not available in our database. This information may pose some bias in our study and further longitudinal researches are needed to validate these results. Secondly, there may have been a high rate of false-positive results for hematuria or proteinuria as a result of the students' physical activity, presence of fever, diet, supine or standing position, and hydration status (37). The specificity of urine tests for abnormal urine examination can be increased with repeat dipstick and/or microscope examinations (38). In the present study, although our urine examination was performed on a single occasion, students with persistent 1+ or higher grade hematuria or proteinuria, in both initial and follow-up testing within the 3-year interval, were selected for analysis. This strategy can elevate the accuracy of our urinalysis. Thirdly, although increasing evidence has revealed that obesity-related glomerulopathy can develop even in childhood (27), our results revealed a non-statistically significant association between persistently overweight students and hematuria or proteinuria. Due to the retrospective nature of the study, cardiometabolic risk factors, such as hypertension, dysglycemia, and dyslipidemia, could not be included in the analysis, which may have created some bias in our study. Further prospective studies to determine associations between these metabolic syndrome components and abnormal urinalysis in obese students should be considered.

## Conclusion

Routine health examination with urinalysis screening for hematuria and proteinuria can detect and predict early-stage chronic renal disease in school children. Our study suggests that children with abnormal BMI should be offered a temporal follow-up from early elementary school age (7–8 years). Our study also highlights the need for long-term prospective observational studies on the risk factors of CKD in adolescents and young adults in Taiwan. In summary, our current study provides useful data for policymakers, primary health care practitioners, and pediatricians to establish necessary future protocols on pediatric CKD prevention in Taiwan.

## Data Availability Statement

The original contributions presented in the study are included in the article/supplementary materials, further inquiries can be directed to the corresponding author/s.

## Ethics Statement

The studies involving human participants were reviewed and approved by The Protection of Human Subjects Institutional Review Board of Tzu Chi University and Hospital. Written informed consent to participate in this study was provided by the participants' legal guardian/next of kin.

## Author Contributions

M-CC, Y-HC, and C-FC conceived and designed the experiments and wrote the paper. M-CC and J-HW analyzed the data. All authors contributed to the article and approved the submitted manuscript.

## Conflict of Interest

The authors declare that the research was conducted in the absence of any commercial or financial relationships that could be construed as a potential conflict of interest.
